# Cell-cell communication enhances bacterial chemotaxis toward external attractants

**DOI:** 10.1038/s41598-017-13183-9

**Published:** 2017-10-09

**Authors:** Zhicheng Long, Bryan Quaife, Hanna Salman, Zoltán N. Oltvai

**Affiliations:** 1 Departments of Pathology, University of Pittsburgh, Pittsburgh, PA 15261 USA; 2 Physics and Astronomy, University of Pittsburgh, Pittsburgh, PA 15260 USA; 3 Department of Scientific Computing, Florida State University, Tallahassee, FL 32306 USA; 4 Department of Computational & Systems Biology, University of Pittsburgh, Pittsburgh, PA 15213 USA

## Abstract

Bacteria are able to coordinate their movement, growth and biochemical activities through cell-cell communication. While the biophysical mechanism of bacterial chemotaxis has been well understood in individual cells, the role of communication in the chemotaxis of bacterial populations is not clear. Here we report experimental evidence for cell-cell communication that significantly enhances the chemotactic migration of bacterial populations, a finding that we further substantiate using numerical simulations. Using a microfluidic approach, we find that *E. coli* cells respond to the gradient of chemoattractant not only by biasing their own random-walk swimming pattern through the well-understood intracellular chemotaxis signaling, but also by actively secreting a chemical signal into the extracellular medium, possibly through a hitherto unknown communication signal transduction pathway. This extracellular signaling molecule is a strong chemoattractant that attracts distant cells to the food source. The observed behavior may represent a common evolved solution to accelerate the function of biochemical networks of interacting cells.

## Introduction

Chemotaxis, the process by which bacterial cells migrate toward favorable chemicals and away from unfavorable ones, is crucial for their survival and growth in natural environments. Since the pioneering work of Adler^1,2^ in the 1960s, the sensory mechanism and the signaling pathway that mediate bacterial chemotaxis have become considerably well understood^2–6^. In a uniform chemical environment bacteria swim in a random-walk pattern, in which the swimming period (run) is punctuated by random reorienting tumbles. In a gradient of chemical cues the frequency of tumbling is reduced when the cell is moving towards the better environment. As a result, bacteria migrate up an attractant gradient or down a repellent gradient in a biased random walk process. During swimming periods, the bacteria are propelled forward by long helical flagella rotated via bidirectional rotary motors embedded in the cell membrane. When the motors rotate counterclockwise, all flagella bundle behind the cell body and push the bacterium forward. In contrast, a clockwise rotation of one or more of the motors, causes the flagella to leave the bundle and therefore a reorientation of the cell body occurs.

The signaling pathway controlling bacterial chemotaxis has been most extensively studied in the model bacterium, *Escherichia coli* K12. These *E. coli* cells sense chemoeffector gradients through five chemoreceptors (*Tsr, Tar, Tap, Trg* and *Aer*) that are clustered at the bacterial poles, of which *Tsr* and *Tar* are the most abundant. These chemoreceptors sense extracellular molecules, primarily amino acids, and utilize a set of cytoplasmic signaling proteins to control flagellar rotation and sensory adaptation^3,6^.

While the chemotaxis sensory system function within individual cells, studies in the past two decades also indicate that bacteria are social organisms and are able to communicate with one another through a variety of chemical signals^7–9^. One of the best-studied cell-cell communication system in bacteria is quorum sensing (QS)^10–13^. Bacteria use QS to regulate gene expression based on the local cell density and in this way coordinate certain behaviors such as virulence, antibiotic resistance, and biofilm formation. QS is mediated by secretion and detection of small diffusible signaling molecules, termed autoinducers. Only when the extracellular concentration of the autoinducer, which increases with the population density, reaches a threshold level do the cells respond to it and alter their gene expression and, consequently, their physiological activities. The autoinducer molecules produced by different species of bacteria are structurally diverse^8,12^. While many Gram-positive bacteria communicate with oligopeptides signals, Gram-negative bacteria frequently use N-acylhomoserine lactones (AHLs) as signaling molecules. *E. coli*, however, is not able to produce AHLs and use instead a furanosyl borate diester, called Autoinducer-2 (AI-2) as the signal^14^.

Besides altering their gene expression, motile bacteria respond to chemical signals by carrying out collective migration and the formation of dense aggregates or complex patterns through chemotaxis. In 1966, Adler had observed that motile *E. coli* cells placed at one end of a capillary containing a mixture of 20 amino acids migrated out in one or two distinct bands^15^. He noted that the formation and movement of the bands were due to local gradients of oxygen and serine that were rapidly depleted by the crowded cells within the bands. On semi-solid agar these travelling bands were displayed in a series of concentric rings (swarm rings) when the cells were placed at the center.

Later, it became evident that bacteria could form more complex patterns on agar plates^16^. For example, Budrene & Berg reported that *E. coli* cells grow into complex arrays of patterns containing rings, spots and stripes on semi-solid agar with selected growth substrates^17,18^. They concluded that formation of these patterns were not due to local depletion of a metabolizable attractants; Instead, the cells aggregated in response to gradients of attractant (aspartate), which they excrete themselves. Later, Park *et al*. found that the aforementioned high density bacterial travelling bands could allow starved *E. coli* cells to find and collapse into confining topologies, e.g., to cluster into the dead ends of a microfluidic maze or collapse into a small square through a narrow opening^19,20^. This behavior is regulated by the *Tsr* chemoreceptor and is a chemotactic response of starved cells to a gradient of attractant that they themselves secrete. By measuring the free amino acids content in the bulk culture media, Park *et al*. concluded that the excreted attractant is the amino acid, glycine, known to be sensed by *Tsr*. In our previous work^21^, we reported a novel collective mode of bacterial dynamics, in which the motile cells rapidly form a sharp band in a microfluidic channel when the cell density reaches a critical value. We have found that this mode is also regulated by *Tsr* and is a result of a positive feedback mechanism provided by bacterial communication.

Compared with communication modes in higher organisms, QS and chemotaxis-based collective behaviors in bacteria are thought to be based on passive communication, i.e., the signal molecules, either the autoinducers or the amino acids, are secreted constitutively through cellular metabolism by all cells in the population. However, the cells respond to the signal only when their concentration reaches a critical threshold or when a noticeable gradient has been established. These processes are thus usually slow and take hours to reach the threshold. It has been hypothesized, however, that bacterial cells might also be able to secrete some signals actively and quickly to rapidly share information on environmental changes with other bacteria^16,22^. This hypothesis is supported by recent experimental work, in which Süel and colleagues have reported that cells of *B. subtilis* biofilms can secrete potassium that lead to the active production of electrical waves that propagate through the biofilm and coordinate the cells’ metabolic states^23^. This potassium ion channel-mediated electrical signaling could also extend beyond the boundaries of the biofilm to attract more distant bacterial cells^24^.

Here, we present additional experimental evidence that bacteria can actively and quickly communicate with each other. Using a microfluidic platform, we show that *E. coli* cells respond to a chemoattractant gradient not only by biasing their own random-walk swim pattern, but also by simultaneously secreting chemical signals into the medium when they sense a steep gradient of their preferred metabolic substrates. This signaling molecule is a strong chemoattractant that promotes the rapid migration of more distant cells toward the food source.

## Results

### Migration of *E. coli* cells in microfluidic chambers following dynamic gradients of attractants

**Figure 1:**
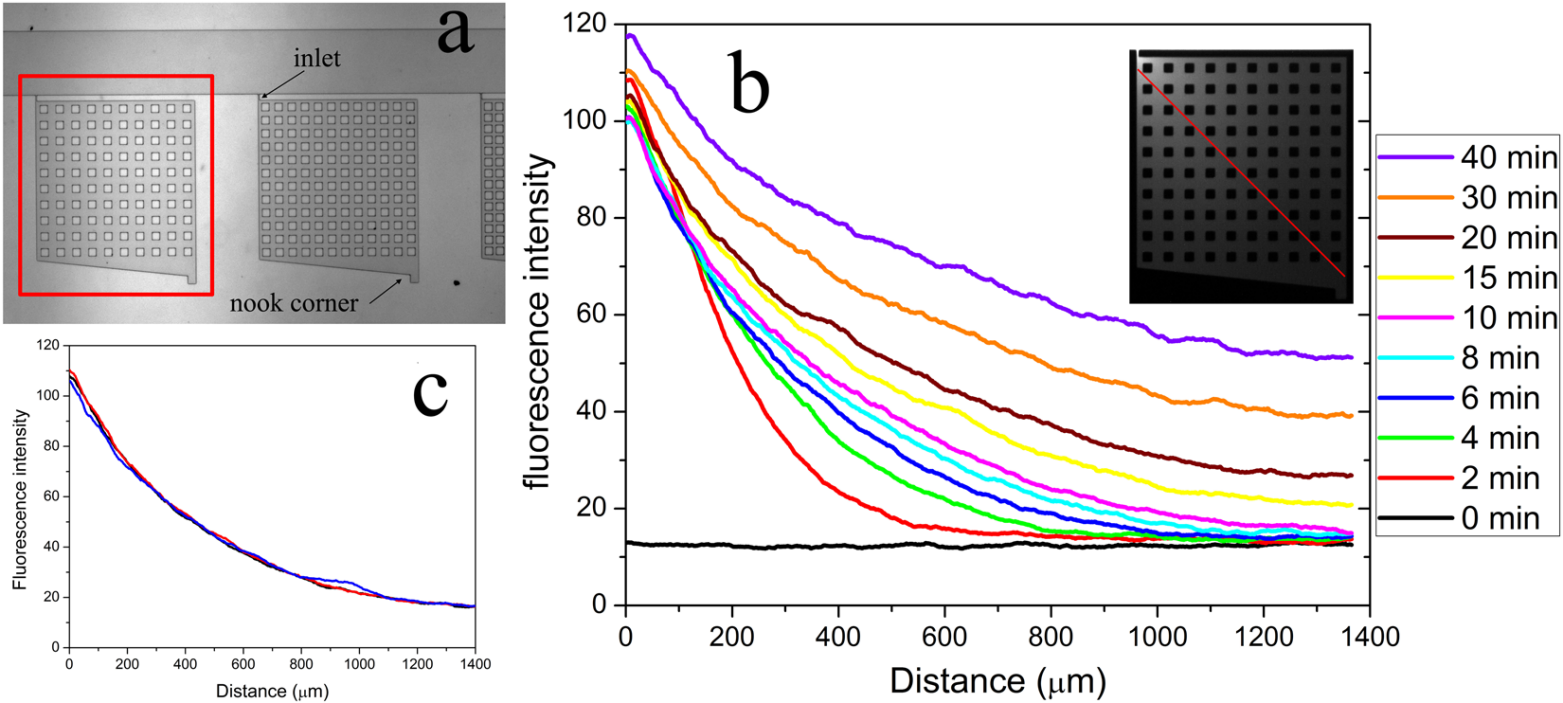
Dynamic gradient inside the microfluidic chambers. (**a**) Partial layout of the microfluidic device. The microfluidic device contains eleven consecutive microchambers that are connected to the main channel (400 μm wide) through a 5 μm-wide and 40 μm-long inlet channel. The microchambers are 1 mm wide and 1 mm long (excluding the nook area) and contain various patterns of evenly distributed micropillars. Only results obtained in the highlighted chamber, in which the micropillars are 50 μm long and 50 μm wide and the space between micropillars is 50 μm, are reported in this study. The depth of all the microchambers and channels is ~10 μm. (**b**) The gradient profiles along the red line marked in the inserted image at various times after the start of flow in the main channel are shown. The microchamber was first filled with (non-fluorescent) M9-G medium containing 10 µM aspartate and YFP-expressing *E. coli* cells; M9-G containing 10 µg/mL Alexa Fluor^®^568 was then pumped into the main channel at a flow rate of 5 μL/min. (**c**) Reproducibility of the gradient profile from three independent experiments and devices is shown at the 10 min time point (other time points show similar reproducibility).

We first designed and created a microfluidic device that contains microchambers with regularly spaced micropillars (Fig. 1a), which allow us to observe the dynamics of bacterial chemotaxis at the population and single cell level. A significant advantage of our design, compared with other microfluidic devices for bacterial chemotaxis^25^, is that only chemotaxis-competent, motile bacterial cells are loaded into the microchambers through the 5-µm-wide interconnect channels. Prior to cell loading, the chambers and the main channel were filled with M9-G medium containing 10 µM aspartate (Asp). We then replaced the medium in the main channel with the cell suspension prepared with the same medium without the addition of Asp. Consequently, a shallow aspartate gradient formed between the chambers and the main channel during cell loading. Motile *E. coli* cells follow this gradient and swim into the chambers by chemotaxis. Thousands of cells could be loaded into the chambers in 5–10 minutes and strong cell accumulation could be observed at the edge of the narrow inlet channel. As seen in Supplementary movie S1, 825 cells were loaded into the left chamber containing 10 µM Asp in 80 seconds. In contrast, in the same time interval only 78 cells swam into the right chamber, which did not contain aspartate, even though the cell density in the main channel was higher. Moreover, in the absence of Asp many non-motile cells get trapped in the inlet corner, which interfere with the subsequent observation and data analysis.

Once the chambers were filled with motile cells distributed evenly within the chambers, we pumped fresh medium containing a chemoattractant into the main channel. In some experiments, a fluorescent tracer dye (Alexa Fluor^®^568 - carboxylic acid) was added to the test medium to allow the simultaneous visualization of the (proxy) gradient of chemoattractant inside the chambers together with the bacterial migration. The gradient of the tracer dye formed in the chamber immediately after the dye solution was pumped into the main channel (Supplementary Fig. S1). While the dye concentration near the inlet remains relatively stable its concentration at the center and at the nook corner increases over time. Consequently, the gradient of the chemoattractants (as approximated by the tracer dye) inside the chambers varies throughout the experiments. Figure 1b depicts the gradient profile of the Alexa Fluor^®^568 tracer dye along a line from the inlet corner to the nook corner at various times. Although the steepness of the gradient decreases noticeably with time, a positive gradient of the tracer dye, -with highest concentration at the inlet corner-, was maintained inside the chamber for more than two hours (while the time-lapse movies in our experiments were typically taken in the first hour). Moreover, the gradient profiles in different devices proved highly reproducible when the flow rates in the main channel were maintained constant (Fig. 1c).

In our microfluidic device, the material exchange between the microchambers and the main channel is only through a very narrow inlet channel. When there is no flow in the main channel, the dye concentration in the microchambers changes much slower (Supplementary Fig. S2) than that in the presence of a flow (Fig. 1b). This implies that the rapid generation of the gradient inside the chambers is not only by diffusion. Instead, at the beginning the flow in the main channel would deform the elastic PDMS chamber ceiling slightly and thus cause a weak flow from the main channel to the chambers. Indeed, we have observed this flow directly in some movies where a number of cells were pushed into the chamber when the flow in the main channel started. The flow seems to be stronger in those chambers without any micropillar support as a result of a larger ceiling deformation. This weak cavity flow helps build the steep gradients at the inlet corner of the chambers in the first few minutes (Fig. 1). By tracking the non-motile cells in the movies, we found that this deformation induced flow diminished in one or two minutes. This indicates the deformed PDMS chamber reached its new equilibrium state rapidly and then the hydrodynamic pressure from the main channel was balanced. As a result, the flow inside the chambers was much slower than the swimming rates of the motile cells and its effect on the cell migration was not noticeable when the flow rate in the main channel was maintained constant. In addition, the flow rate in the main channel was the same in all experiments reported here, and as a result the hydrodynamic pressure is similar in all experiments. Therefore, the different behaviors observed under different experimental conditions cannot stem from hydrodynamic effects.

**Figure 2:**
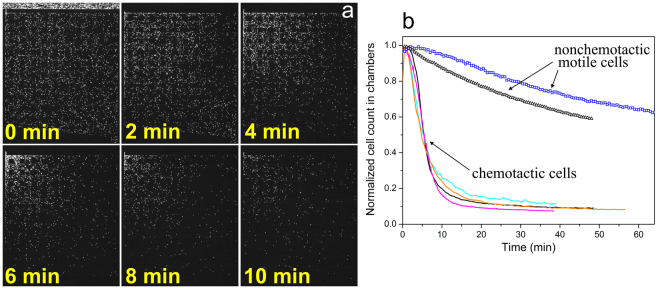
*E. coli* RP437 cells display rapid migration in response to aspartate gradients. (**a**) Time-series snapshots from movie S1 show that YFP-expressing *E. coli* cells migrate toward the inlet corner in the upper left (where the concentration of Aspartate is higher) and then escape through the inlet to the main channel; (**b**) The cell count in the microchambers (normalized to the maximum cell count in the chambers) decreases with time. The solid and scatter curves show the escape rate of wild-type cells in M9-G medium with or without methionine, respectively. Each curve have been obtained from independent experiments (shown in different colors) performed on different days and shows the averaged result from 2–3 movies in the same experiment. The chambers were filled with M9-G (with or without methionine) containing 10 µM Asp before loading the cells. Fresh M9-G containing 200 μM Asp (except for the orange curve, which contains 20 μM Asp) was pumped into the main channel after loading.

Figure 2a and Supplementary movie S2 show a typical migration behavior of wild-type (*wt*) *E. coli* RP437 cells inside the chambers after the chemoattractant (200 µM Asp) was pumped through the main channel. Immediately after the gradient of attractant formed in the chambers, cells started to migrate toward the inlet corner with higher aspartate concentration and then escaped into the main channel through the narrow inlet. Figure 2b shows the total cell count in the chambers as a function of time. It is evident that the migration rate of the chemotactic cells is fast and about 80% of the cells escape from the microchambers in the first ten minutes. This rapid migration is mainly due to chemotaxis, as cells escaped at a much slower rate when the M9-G medium did not contain the chemotaxis-essential amino acid, methionine, which renders the cells practically non-chemotactic (Fig. 2b).

### Rapid cell migration is a chemotactic response that is enhanced by cell-cell communication

To evaluate the potential contributions of other cellular behaviors to the observed rapid cell migration, we next examined the migration dynamics of wild-type (*wt*) *E. coli* and its isogenic *Δtsr* and *Δtar* mutants, which lack the *Tsr* and *Tar* chemoreceptors, respectively. Chemotaxis of individual bacteria in an aspartate gradient is mediated by the Tar receptor^3^. Many studies have shown that deletion of *Tsr* does not affect the chemotaxis capability of *E. coli* cells in Asp gradient^3,26,27^. Indeed, we could rapidly load *Δtsr* mutant cells into the microchambers (containing 10 µM Asp) at a similar speed to *w*t cells, while loading of *Δtar* mutant cells under the same conditions proved very difficult. In separate experiments, we have also found that both *wt* and *Δtsr* cells migrate toward the nook corner at similar speed in a reverse gradient (with highest concentration at the nook) of aspartate. The reverse gradient was created by first filling the chamber with M9-G medium containing 100 µM Asp and then pumping fresh M9-G (without the addition of aspartate) through the main channel after cell loading (data not shown). These results confirm that the *Δtsr* cells used in this study are capable of migrating up the aspartate gradient.

**Figure 3:**
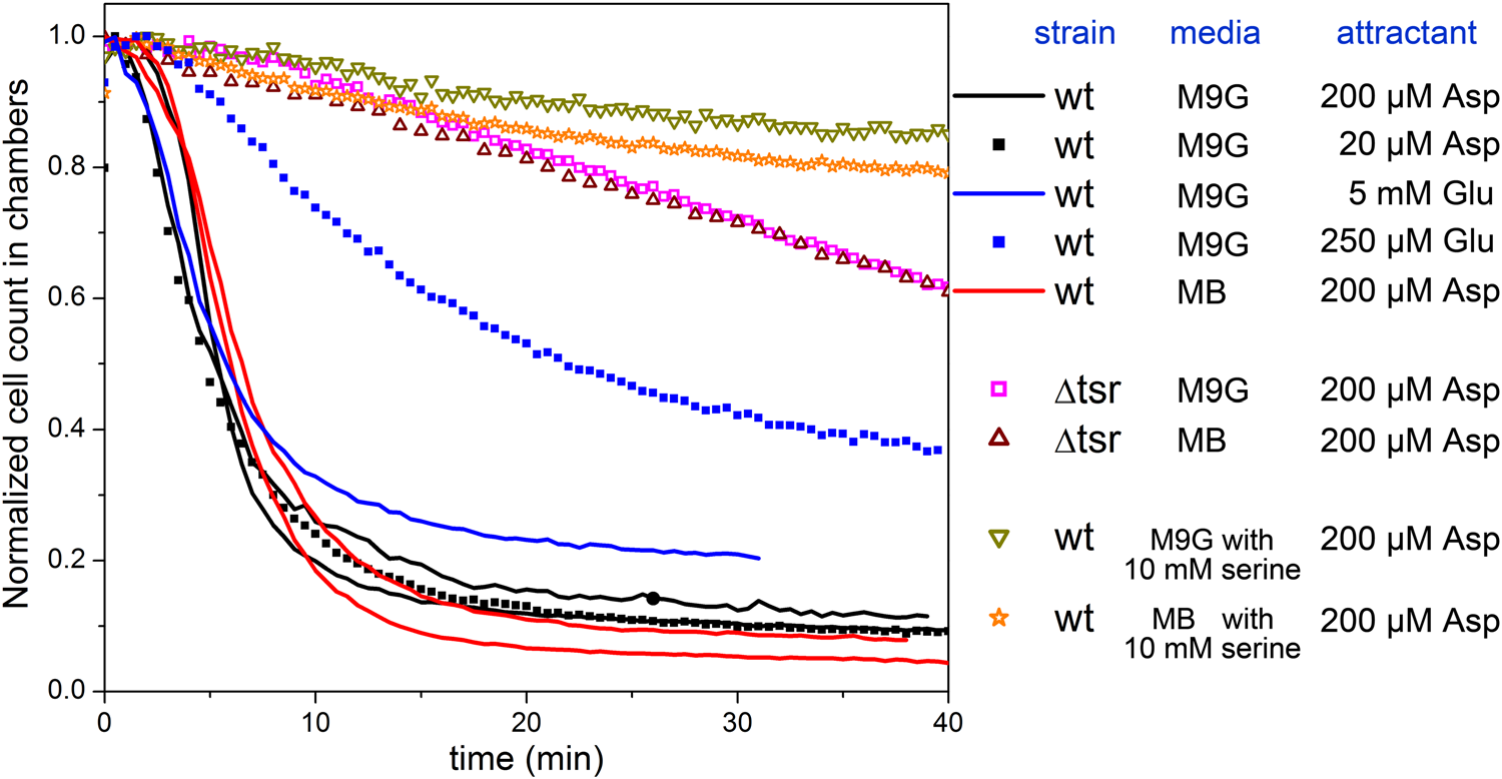
Migration rate of the wild-type and *∆tsr* mutant cells in M9G and motility buffer. The results were obtained from independent experiments performed on different days on the indicated *E. coli* strains. Each curve shows the averages of 2 or 3 movies in the same experiment. The solid curves obtained from the experiments with the same strain and the same media are displayed in the same color. The chambers were filled first with M9-G or motility buffer (MB) containing 10 µM Asp to facilitate the cell loading. Fresh M9-G or MB containing various concentration of aspartate (Asp) or glutamate (Glu) was pumped into the main channel after loading. For the saturation experiments, 10 mM serine was added in M9-G or MB and was used throughout the experiment, except in the media in the culture tubes.

Surprisingly, despite the fact that aspartate only engages the Tar receptor, *Δtsr* mutants migrate much slower than *wt E. coli* cells in aspartate gradients in M9-G and motility buffer (MB) (Fig. 3 and Supplementary movie S3) (note that *Δtsr* mutants display a similar behavior in the complex gradient of M9CG (Supplementary Fig. S3) where the gradient was created by the bacterial cells’ rapid consumption of the amino acids in the microchambers). Moreover, the rapid migration of *wt E. coli* cells was also suppressed by the addition of 10 mM L-serine into the testing medium that is known to saturate *Tsr* ^19,21,26^. Also, *wt* *E. coli* cells are very sensitive to the Asp gradient; applying 20 µM Asp in the main channel elicits a similarly strong response as seen with 200 µM Asp (Fig. 3). However, the cells are less sensitive to the Tar-specific chemoattractant, glutamate (Glu) gradient; the migration rate with 5 mM Glu is comparable to that in Asp gradient, while the migration toward 250 µM Glu is significantly slower but still faster than that of the *Δtsr* cells (Fig. 3). These results imply that the Tsr chemoreceptor is required for the rapid migration of *wt E. coli* cells in response to the gradient of Asp or Glu. Therefore, the difference in migration behavior between *wt* and *Δtsr* cells suggests that the observed rapid migration of *wt* cells is not only in response to aspartate or glutamate.

Bacteria can communicate with one another through various excreted signaling molecules. The observed rapid migration could reflect a chemotactic response enhanced by signaling molecule(s) that *E. coli* cells use to share the information about their environment with each other. We have thus hypothesized that *E. coli* cells secrete a signaling molecule when sensing a steep gradient of favored food. The cells farther away would then follow the gradient of the signaling molecule and migrate toward the food source. Moreover, our data indicate that the signaling molecule is sensed by the *Tsr* receptor; *Δtsr* cells are still able to sense the gradient of aspartate and secrete the signaling molecule, but they cannot sense it because they lack the *Tsr* receptor. Thus, the migration of *Δtsr* cells inside the chamber could be attributed to chemotaxis toward aspartate, while the rapid migration of *wt* cells may be mainly due to chemotaxis toward the bacteria-secreted signaling molecule(s).

**Figure 4:**
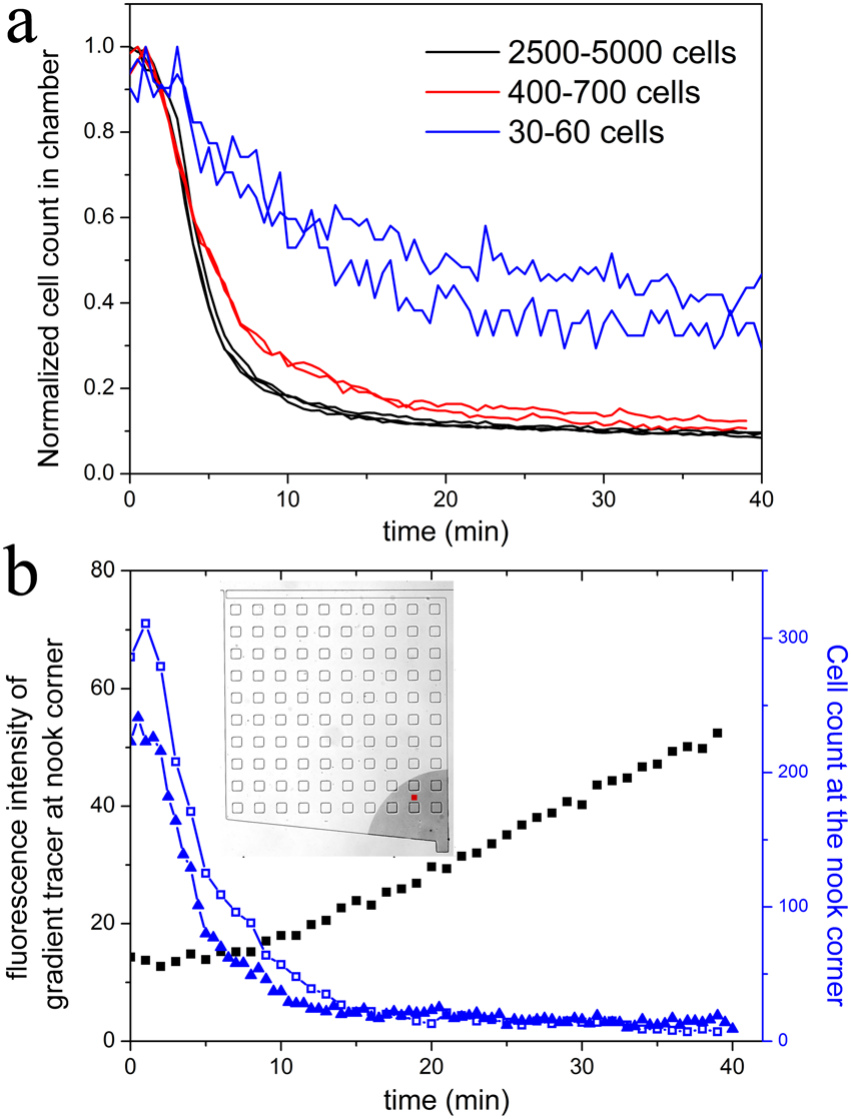
Effects of cell-cell signaling on the migration of bacteria out of the microchambers. (**a**) The effect of cell densities in the microchambers on the escape rates of *wt* cells is shown. The chambers were filled with M9-G medium containing 10 µM Asp before being loaded with various densities of *wt* cells. Fresh M9-G medium containing 200 μM Asp was pumped into the main channel after loading. (**b**) The dynamics of gradient and cell count at the nook corner. The black curve shows the change of the fluorescence intensity of the Alexa Fluor^®^568 tracer at the red position showed in the inset and Supplementary Fig. S1. The blue curve shows the cell count within the 300 pixel range (shaded area in the insert) from the nook corner. The chambers were filled with M9-G containing 10 µM Asp before loading the cells. Fresh M9-G containing 200 μM (open square symbol) or 20 μM (solid triangle symbol) Asp was pumped into the main channel after loading. The wild-type cells at the nook corner start migrating toward the inlet before the positive gradient of the tracer dye (a proxy for Asp gradient) is established.

This hypothesis is supported by the following observations: First, we investigated the effect of cell density on the escape rates of *wt* *E. coli* cells by varying the population size of cells in the microchambers. As shown in Fig. 4a, at high cell density *wt* *E. coli* cells migrate out of the chamber at a faster rate than at low cell density. This density dependency suggests that the observed rapid migration (defined as a migration rate with ≥50% of the cells escaping from the chamber in 10 minutes) of *wt* cells in Asp gradient is not in response to the external chemotaxis signal alone. The effect of the external signal is independent of the population size. However, the concentration of excreted signaling molecules would be proportional to the population size. Therefore, when the population is small its effect is diminished, as demonstrated here.

Additionally, our measurements of the tracer dye gradient show that it is very shallow at the nook corner in the first six minutes of the experiments (Fig. 1). The fluorescence intensity of the tracer dye at the spot in the nook corner (Fig. 4b and Supplementary Fig. S1) remains steady in the first eight minutes. Although the Alexa Fluor^®^568 dye has different molecular weight from aspartate, their diffusivities in aqueous solution are comparable. The diffusion coefficients of the Alexa Fluor dye and aspartate were reported as 3.4 × 10^−10^ m^2^ s^−1^ and 5.5 × 10^−10^ m^2^ s^−1^ at 25 °C, respectively^25,28^. Also, as mentioned, the gradient inside the chambers formed not only by diffusion but also largely by the cavity flow caused by the chamber deformation. Thus the gradient of the tracer dye could represent that of many low-molecular-weight chemoattractant such as aspartate. In our experiments, the medium inside the chambers already contains 10 µM Asp before loading. Based on Supplementary Fig. S2, the residual Asp concentration at the nook corner after 30 min loading is estimated to be ~8.5 µM. However, the Asp concentration at the same spot in Fig. 4b and Supplementary Fig. S1 is lower than 5 µM (assuming that the medium in the main channel contains 200 µM Asp and no Asp in chamber at the beginning) in the first 10 minutes. Therefore, it appears that a positive gradient of aspartate is not being established at the nook corner in the first 10 minutes after the medium containing 200 µM Asp is pumped through the main channel. Yet, we find that the most distant cells in the nook corner start migrating toward the inlet starting less than two minutes after applying the testing medium through the main channel, and presumably before they sense the positive Asp gradient. As seen in Fig. 4b, more than two thirds of the cells have migrated out of the nook corner in eight minutes, even when the concentration of the Asp in the main channel is only 20 µM (while it takes more than 60 minutes to build a positive gradient in the nook corner). These results suggest that the initial rapid migration of distant cells at the nook corner (i.e., those farthest away from the inlet) is not in response to the Asp gradient. Instead, the cells appear to migrate toward the inlet corner by following the gradient of a signaling molecule secreted by the cells in the rest of the chamber, where the gradient of aspartate formed rapidly. The rapid migration of distant cells indicates that the putative signaling molecule is able to exert its effect over a long range.

**Figure 5:**
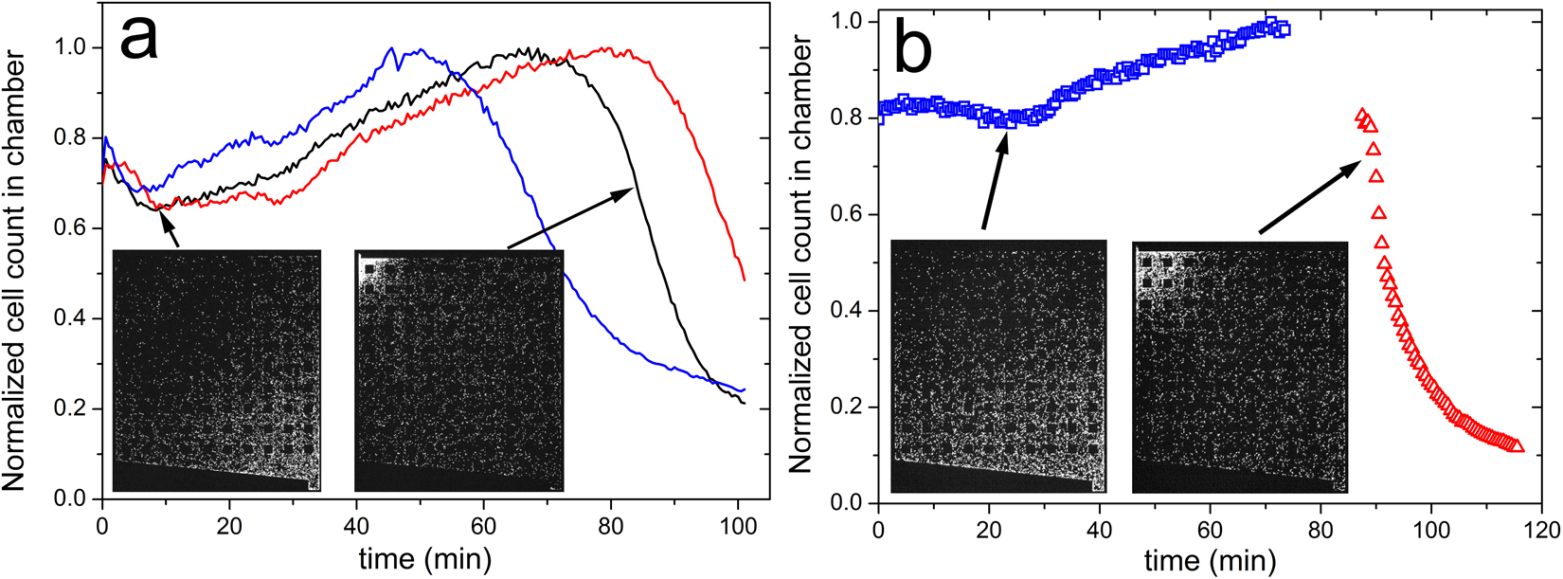
Chemotaxis competition between aspartate and the signaling molecule. (**a**) The signaling molecule is a stronger attractant than Asp. The chambers were filled with M9-G with 10 µM Asp prior to the loading of *wt* cells, followed by loading more than 5,000 cells into the microchambers. Fresh M9-G containing 200 μM Asp was pumped into the main channel when the cells start migrating toward the nook corner. The curves in different colors were obtained from three different chambers in the same experiment. The two inserted images are two snapshot from movie S4 at 10 min (left inset) and 85 min (right inset), respectively. It is likely that when cells accumulate at the nook corner they follow a cell-cell communication-generated signal and not the Asp gradient. (**b**) Serine is a stronger attractant than aspartate. The chambers were filled with M9CG before loading the cells. Fresh M9-G containing 300 µM Asp (blue symbol) was firstly pumped into the main channel after loading. The media in the main channel was then changed to M9-G containing 10 µM serine (red symbol). The two inserted images are two snapshot at 24 min (left inset) and 89 min (right inset), respectively.

Park *et al*. previously reported that high density of chemotactic *E. coli* cells could find and accumulate in confining regions in a maze^19,20^. By loading more than 5,000 cells into the chambers, we observed that *wt E. coli* cells could accumulate at the nook corner when we increased the loading time. In such experiments, the *wt* cells first accumulated at the nook corner, presumably in response to a secreted signaling molecule that mediate cell-cell communication, and did not migrate out of the chamber after 200 µM aspartate was pumped into the main channel (Fig. 5a and Supplementary movie S4). If the cells first aggregate at the nook corner, a reverse gradient of the signaling molecule is presumably established in the chamber, with the highest concentration being at the nook. The migration toward the nook area instead of toward the high concentration of Asp indicates that the putative signaling molecule(s) is a stronger chemoattractant than aspartate. In this experiment, the cells that accumulated at the nook corner dispersed after 40 minutes and the reverse gradient of the signaling molecule diminished gradually. When more dispersed cells got to the inlet corner, the steep gradient of aspartate near the inlet may have triggered them to secrete more signaling molecule and thus the cells migrate out of the chamber rapidly at last.

### Attempts to identify the signaling molecule

The above results indicate that the signaling molecule is a strong chemoattractant that is sensed by the *Tsr* chemoreceptor. In an attempt to identify the signaling molecule, we next tested two potential molecules that have been thought to play important roles in bacterial collective behaviors and in bacterial quorum sensing.

The first candidate molecule we tested was the amino acid, glycine, which is known to be sensed by *Tsr* ^3,27^. In our first experiment, we loaded wild-type cells into the microchambers containing M9-G and 10 µM aspartate, then used M9-G containing 100 µM glycine as the attractant in the main channel. We found that the cell number inside the chamber did not change much in the gradient of glycine (Supplementary Fig. S4a). However, the same cells migrated out of the chamber rapidly once the medium in the main channel has been replaced with M9-G containing 200 µM aspartate (Supplementary Fig. S4a). In a second experiment, the cells were loaded into the chambers containing M9CG and 10 mM glycine and then fresh M9CG was pumped into the main channel. We found that high concentration of glycine inside the chamber did not suppress the rapid migration of wild-type cells (Supplementary Fig. S4a). These results suggest that glycine in itself is not the signaling molecule that initiates the rapid cell migration.

Next, we examined the potential chemotaxis-enhancing role of the signaling molecule, autoinducer-2 (AI-2) that plays a key role in quorum sensing and biofilm formation in *E. coli* ^11,12,14^. AI-2 is produced by the enzyme, S-ribosylhomocysteine lyase, (encoded by the *luxS* gene) and is also a chemoattractant for *E. coli*, a process that is mediated by the AI-2 binding protein, LsrB, and by Tsr receptor^29^. We tested *∆luxS* and *∆lsrB* mutant *E. coli* strains in the same conditions as that for *wt* cells (the *∆luxS* strain is unable to produce AI-2 and the *∆lsrB* strain cannot sense and respond to the gradient of AI-2). As seen in Supplementary Fig. S4b, both strains migrated out of the chamber rapidly in the gradient of aspartate. This indicates that the putative signaling molecule is not likely to be AI-2 alone.

We also analyzed the free amino acids in the extracellular medium by HPLC. Since the concentration of the secreted amino acids in fresh M9-GA (not containing any amino acids except 100 µM Asp and 20 µM methionine) is very low, the experiments in the microchambers were scaled up to 5 mL in Falcon centrifuge tubes. As seen in Supplementary Fig. S5, only low concentration (~1.5 µM) of serine was detected in that sample in which the high density (similar to that in the microfluidic setting) of cells were exposed to a dynamic Asp gradient for 20 minutes. In comparison, the serine concentration in the sample without Asp gradient is much lower. Only ~0.8 µM serine was detected in the gradient-absent control sample in which the cell suspension was kept still for 4 hrs and no serine was detected in the control samples with standing time less than 2hrs. Meanwhile, low concentration of a few other amino acids, including glutamate, alanine, tyrosine, valine and an unknown amino acid analogue (small peak at 3.75 min) were also detected in the 120 and 240 minute-standing control samples, indicating that the cells were able to secrete many amino acids slowly through cellular metabolism.

### Simulation of bacterial chemotaxis in the presence of amplifying signal

**Figure 6:**
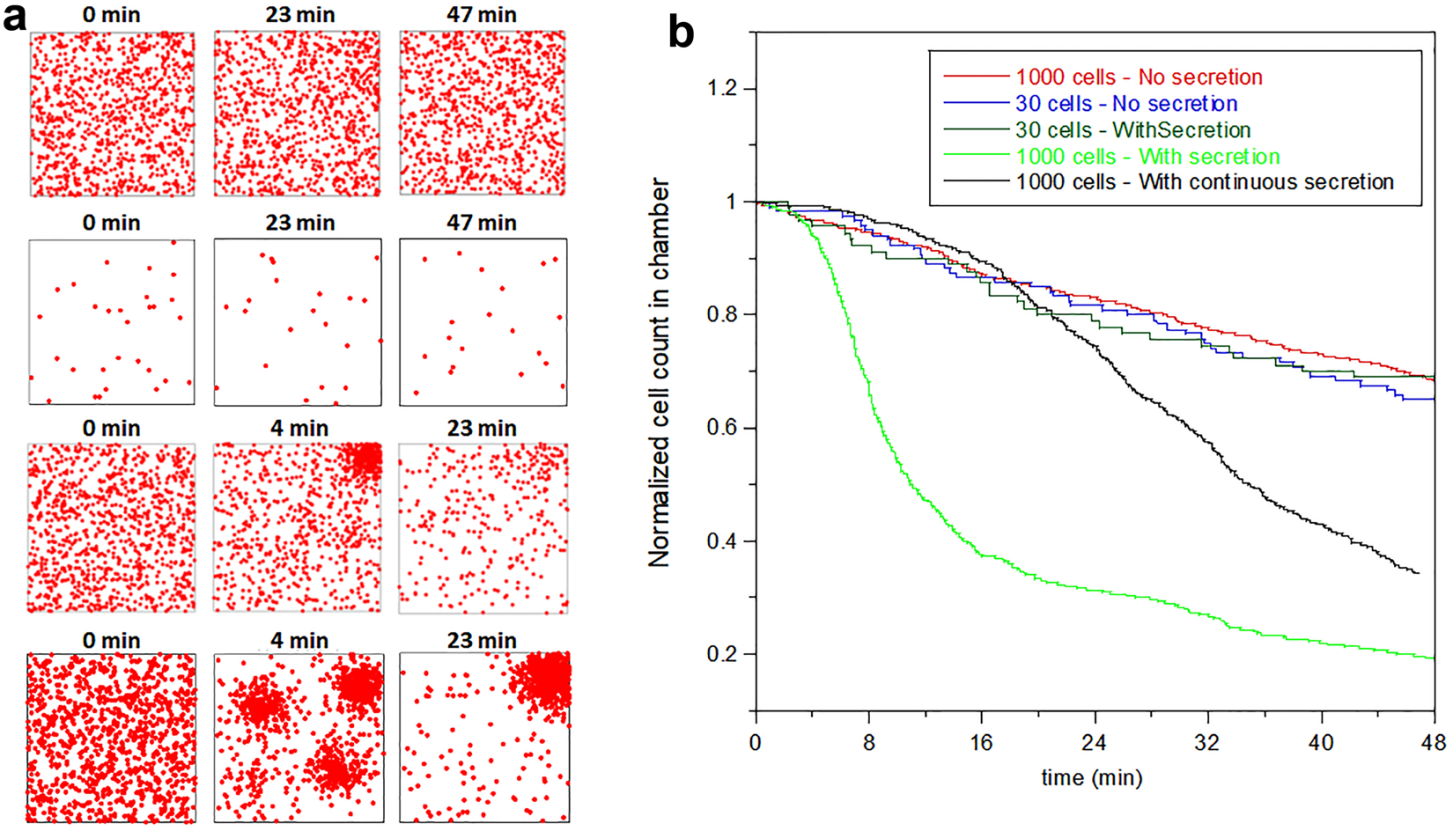
Cell-cell signaling accelerates the exit rate of bacteria from the chamber in numerical simulations. (**a**) Time-series snapshots from movies S5, S6, S7, S8 and S9 show that cells migrate toward the upper right corner (where the concentration of the external attractant is highest) and get trapped there in all tested conditions: high initial cell density without secretion of attractant (NS) (top row), low initial cell density with secretion in response to sensing the external attractant gradient (PS) (second row from top), high initial cell density with secretion of attractant in response to sensing the external attractant gradient (PS) (third row from top), and high cell density with continuous secretion of attractant (FS) (bottom row). (**b**) Fraction of cells remaining in the microchambers as a function of time, obtained from the simulations of the various conditions tested, as described in the legend. The model predicts that the secretion of attractant increases the escape rate of cells from the chamber by attracting the distant cells at the bottom left corner faster towards the external attractant source (at the upper right corner).

To further support the validity of our conclusions, we followed the dynamics of cells escape out of a 1mm × 1mm chamber in numerical simulations, in which cells (or agents) follow an external attractant gradient (see Methods for details of the simulations). We carried out the simulations assuming a homogenous random distribution of bacteria in the microchamber at the start, and examined the following scenarios: 1) bacteria do not secrete a chemotaxis-amplifying signaling molecule; 2) bacteria secrete a chemotaxis-amplifying signaling molecule irrespective of their direction of movement; and 3) bacteria secrete a chemotaxis-amplifying signaling molecule only when swimming in the direction of the chemotaxis gradient. Also, we compared low (30) or high (1,000) cell number at the start of the simulation (Fig. 6).

As depicted in Fig. 6, our simulations indicate that secretion of a chemotaxis-dependent signaling molecule would substantially accelerate the migration of cells up the external gradient and their escape out of the chamber in response to a chemotaxis gradient. Interestingly, the model also predicts that when the secretion is in response to sensing an increase in the external attractant concentration, the escape rate is much faster than in the case where the secretion is independent of the direction of the bacterial movement. This appears to be due to local aggregations of cells created at various locations in the chamber (see Fig. 6a, bottom row at 4 min), which delay the movement of cells towards the external attractant source.

## Discussion

**Figure 7:**
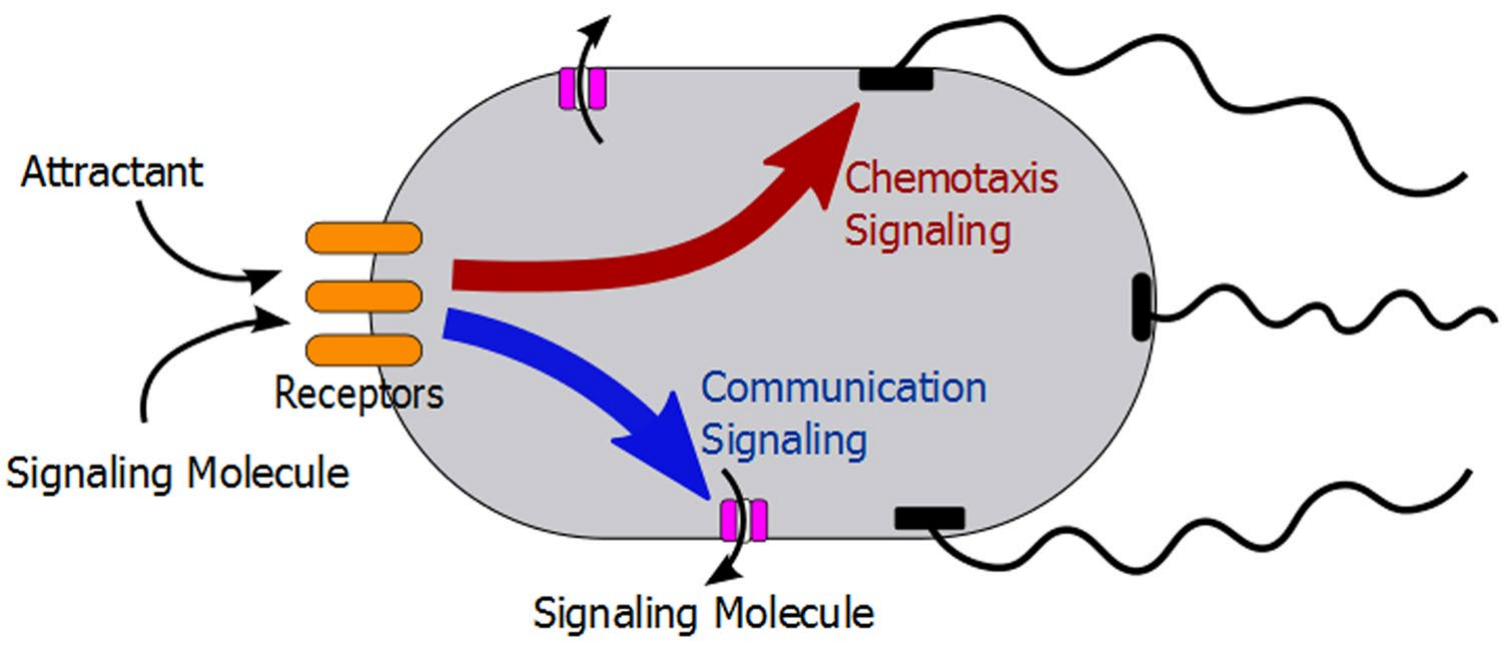
Model of chemotaxis and communication signaling in *E. coli*. The red arrow represents the well-understood chemotaxis signaling pathway. The attractant gradients are first sensed by chemoreceptor and further transduced via a set of cytoplasmic signaling proteins to the flagella motor. The blue arrow stands for the hypothesized communication signal transduction pathway, by which bacterial cells actively secrete a signaling molecule into surrounding medium when sensing the steep gradient of their preferred metabolic substrates.

Our study suggests that *E. coli* cells respond to the chemoattractant gradient not only by biasing their own random-walk-like swimming patterns to migrate up the gradient, but also by simultaneously secreting a chemical signal into the medium. This diffusible signaling molecule is a strong chemoattractant, which then attracts distant cells to the food source by establishing an additional steeper gradient over short distances. The overall interpretation of our results is summarized in a simple model (Fig. 7). We hypothesize that external chemoattractant engaged chemoreceptors simultaneously activate flagellar motion and a hitherto unknown signal transduction pathway by which *E. coli* regulate the active secretion of the signaling molecule into surrounding medium. Since the signaling molecule is a strong chemoattractant, nearby *E. coli* cells sense this diffusible signal and release more signaling molecule into the medium to create an active signal propagation in the population. As a result, *E. coli* cells at the distant nook corner migrate toward the inlet rapidly by following the gradient of the signaling molecule, even before they sense the gradient of the chemoattractant food source (e.g., aspartate).

This rapid and long-range signal propagation implies that secretion of the signaling molecule is unlikely to depend on the gene expression and cell growth. Indeed, we have also observed the rapid migration of the *wt E. coli* cells in the nutrient-free motility buffer (containing 10 µM methionine) (shown in Fig. 3). This result indicates that the signaling molecule is not a direct metabolite of the nutrient source in the testing medium. Instead, the signaling molecule is likely released from an intracellular signal pool through gateable pores in the cell membrane.

Also, we found that the amount of the secreted signaling molecule likely depends on the strength of the chemical stimuli. In our experiments, the migration rate with 20 µM Asp is similar to that with 200 µM Asp. However, only high concentration (5 mM) of glutamate could elicit a similarly strong response to that seen with Asp gradient, while the migration rate with 250 µM Glu are considerably slower (Fig. 3). Since the observed rapid migration is mainly a chemotactic response to the secreted signaling molecule, the slower migration rate in Glu gradient suggests less signaling molecules are secreted into the medium. The difference of the migration rate in Asp and Glu is consistent with the different affinity of the *Tar* receptor to Asp and Glu, suggesting that the putative communication signaling transduction pathway might share the same transmembrane (chemo)receptors with the well-understood chemotaxis signaling pathway (illustrated in Fig. 7). This dependency might also explain the slow self-congregation observed in Fig. 5a and by Park *et al*.^19^. When high density of *wt* cells are placed in a microchamber/microchannel, the rapid consumption of the nutrients by the cells would create local gradients around the cells. These local gradients are then sensed by the cells and trigger the secretion of the signaling molecule. However, it takes tens of minutes to hours for the cells to form the initial aggregate because the direction of the local gradients is random and the concentration of the signaling molecule induced by these local gradients is expected to be low.

Although we have not identified the exact chemical structure of the signaling molecule, we hypothesize that serine is the likely candidate based on: (i) serine is a stronger attractant than aspartate for the *wt* cells used in our study (Fig. 5b). This agrees with the results in Fig. 5a, where *wt* cells migrate toward the signaling molecule instead of Asp; (ii) the chemotaxis toward serine is exclusively mediated by *Tsr* chemoreceptor. The *∆tsr* cells, as well as the *wt* cells in the testing media containing high concentration of serine, are thus not able to follow the signaling molecule and migrate out of the chamber rapidly, in agreement with our experimental observation (Fig. 3); (iii) low concentration of serine was detected in our HPLC analysis of the extracellular media, which did not contain serine at the beginning. *E. coli* cells require large amounts of L-serine to support cell growth. It has been reported that serine is the first amino acid consumed by *E. coli* in complex medium^30^. Therefore, the extracellular serine concentration is expected to be very low even though the cells synthesize a lot of serine and secrete a small portion into the surrounding medium as the signaling molecule for cell-cell communication. Of note, this hypothesis does not conflict with the observation by Park *et al*.^19^, in which they found the glycine concentration in the culture media increased with time after the optical density reached 0.2, and they thus attributed the self-congregation of chemotactic cells into confining topologies to the secretion of glycine instead of serine. Because serine is the amino acid consumed first by *E. coli* cells in complex medium and is the metabolic precursor of glycine, the secreted serine may be consumed rapidly by the cells and converted to glycine leading to an increase of glycine in the growth medium.

In conclusion, our results suggest a hitherto unrecognized mechanism by which chemotaxis is enhanced by signals secreted by communicating *E. coli* cells. While shown here only for bacterial chemotaxis this behavior may represent a common evolved solution to accelerate the function of biochemical networks of cells communicating with one another.

## Materials and Methods

### Bacterial strains and growth conditions

The wild-type *E. coli* K12 strain RP437 and its mutant derivatives HCB317 (*Δtsr*) and RP2361 (*Δtar*) were used for the main part of this study. All strains expressed yellow fluorescent protein (YFP) constitutively under the control of the *λ*-phage pR promoter from the plasmid PZA3R-YFP containing chloramphenicol resistance. In addition, MJ101 (Δ*lsrB*)^29^ and KX1485 (Δ*luxS*) strains derived from the CV1 strain (equivalent to RP437) and expressing green fluorescent protein (GFP) from the plasmid pCM18 containing erythromycin resistance, were used for control tests.

Strains were first grown overnight at 30 °C with agitation at 240 rpm in M9 minimal medium supplemented with 1 g/L casamino acids and 4 g/L glucose (M9CG) and appropriate antibiotics. The overnight cultures were then diluted 100 fold in fresh M9CG and grown at 30 °C until early exponential phase, optical density at 600 nm (OD_600_) of 0.1–0.2. Prior to loading the cells into the microfluidic device (see details below), the cultures were centrifuged, washed, and the cells were resuspended in fresh testing medium. The media used during the experiments in the microfluidic device were either M9CG, M9-G (M9 minimal medium supplemented with 4 g/L glucose and10 µM L-methionine) or motility buffer (MB: 10 mM potassium phosphate, 10 mM sodium lactate, 0.1 mM EDTA, and 10 µM L-methionine, pH = 7.0).

### Microfluidic device

We designed and fabricated a microfluidic device (Fig. 1a) containing eleven consecutive microchambers that were connected to a wide channel through a 5 μm-wide and 40 μm-long inlet channel to allow for the introduction of *E. coli* cells and media into the microchambers. The microchambers were 1 mm wide and 1 mm long (excluding the nook area that we designed for the purpose of other type of experiments) and contained evenly distributed micropillars.

The microdevice was fabricated by standard soft lithography. Briefly, 10 μm-thick photoresist SU-8 2010 (MicroChem, Newton, MA) was spin-coated onto a clean silicon wafer. The wafer was then exposed to UV light through the photomask by Karl Suss MJB3 aligner. After removing the unexposed resist in SU-8 developer, the resulting positive reliefs of the microchannels and the microchambers on the wafer served as a master mold. A 10:1 w/w mixture of the silicon elastomer PDMS prepolymer and its curing agent (Sylgard 184 set, Dow Corning) was then poured over the mold and cured at 65 °C overnight. After curing, the PDMS replicates were peeled off from the mold and bonded to a glass slide by brief treatment in air plasma.

Immediately after the plasma treatment and bonding, 5 μL of 20 mg/mL bovine serum albumin (BSA) solution was added into the two inlet/outlet holes of the PDMS device and kept at room temperature for one hour to coat the walls of the PDMS device with a thin layer of BSA to prevent cell adhesion. We then replaced the BSA solution in the microchannels and microchambers with one of the testing media prior to loading the cells. 10 μM L-aspartic acid (Asp) was usually added to the testing medium to facilitate the cell loading into the microchambers. Motile *E. coli* cells that were resuspended in fresh testing medium (without Asp) were then introduced into the main channel and allowed to swim into the microchambers continuously. After reaching the desired cell density in the microchamber (typically 2,000–3,000 cells in each chamber), fresh testing medium containing certain attractant was pumped through the main channel at a flow rate of 5 μL/min using a syringe pump (Pump Systems Inc.).

### Time-lapse imaging and data analysis

The migration of YFP (or GFP) -expressing *E. coli* cells in the microchambers were observed and recorded in fluorescence mode using a fully automated inverted microscope (Zeiss AxioObserver Z1), equipped with a motorized *x-y* stage (Applied Scientific Instruments). Time-lapse movies were acquired at a rate of 2 frames/min (or 1 frame/min when the gradient tracer was added in the media) using a CCD camera (Zeiss AxioCam MRm) at room temperature (~26 °C). The movies were processed for analysis and display with ImageJ software. The cells inside the microchambers in each image were counted automatically by a custom pipeline with the open-source software, Cellprofiler.

### Free amino acids analysis of extracellular medium

WT RP437 cells were grown in M9CG to OD_600_ = 0.15 in a 1000-mL flask with relatively slow agitation (50 rpm) (to prevent potential shearing of flagella from motile *E. coli* cells). The cells were then washed once in fresh M9-GA (M9 minimal medium supplemented with 4 g/L glucose, 100 µM Asp and 20 µM methionine) by centrifugation and resuspended in 30 mL fresh M9-GA (OD = 0.55). The cell suspension was then divided into two halves. The first 15 mL suspension was kept at room temperature and used as controls in the absence of Asp gradient. 5 mL aliquots were removed at 1, 120 and 240 minutes after resuspension. For the secretion analysis in the Asp gradient, 5 mL each of the cell suspension was transferred into three 10 mL Falcon centrifuge tubes. 1 mM Asp was then slowly pumped into the upper layer of each tube at a flow rate of 4 μL/min for 5, 20 and 40 minutes, respectively.

The cells in the samples were removed immediately by centrifugation and the supernatants were filtered, frozen and sent to the Washington University’s Danforth Plant Science Center Proteomics & Mass Spectrometry Facility (St. Louis, MO) for analysis. There, the samples were dried with speed vacuum, and they were then extracted as follows: 600 μL of water: chloroform: methanol (3:5:12 v/v) extraction solvent was added the supernatant were collected and the residues were re-extracted with another 600 μL of water: chloroform: methanol extraction solvent. Supernatants were combined and then 300 μL of chloroform and 450 μL of water were added. The upper phase was collected and dried. The pellets were resuspended in 50 μL of 20 mM HCl. During derivatization, the samples were diluted ten times; 10 μL was used for a reaction volume totaling 100 μL, and 1 μL was injected for the reaction.

### Numerical simulations

The simulations were carried out using Matlab (MathWorks). Each simulation run starts with a homogenous random distribution of bacteria in the chamber. The attractant concentration (*c*) at the location of each bacterium is then calculated and each bacterium is assigned a random run direction (*θ*) in the 2-d space. For each bacterium, a speed (*s*) and a total run time (*T*) are chosen from a Lognormal distribution and a Poisson distribution, respectively. The run of all bacteria is then initiated with the parameters assigned. While the run time (*t*) is less than the total run time (*T*), the positions of the bacteria are updated using:

1 $$x(t+{\rm{\Delta }}t)=x(t)+s{\rm{\Delta }}t\,\cos (\theta )$$

2 $$y(t+{\rm{\Delta }}t)=y(t)+s{\rm{\Delta }}t\,\sin (\theta )$$

If a bacterium reaches one of the domain boundaries before the total run time (*T*) is completed, a new speed and direction are assigned to it such that it moves back into the computation domain. The bacterium then continues its run until *T* is completed. Once the total run time is completed, the attractant concentration at the new location of each bacterium is computed. In the case where the bacteria secrete more attractant, the location of each bacterium and the time it arrived there are saved so that the diffusion equation for the secreted attractant can be solved in order to include it in the calculation of the attractant concentration other bacteria sense at that location in future time. The concentration of attractant at the new and old positions of each bacterium are used to calculate the change in attractant concentration:

3 $${\rm{\Delta }}c=c({t}_{2})-c({t}_{1})$$

The value of Δ*c* is used to determine the probability of direction change for each cell given by the step function:

4 $$P=\frac{0.75}{1+exp({\rm{\Delta }}c-5)}+0.25$$

This function in principle, causes the cells to always change direction if the change in attractant concentration experienced by the cell is less than a certain threshold. When the concentration change is above that threshold, the probability to change direction decreases to 0.25, which allows the cell to extend its run in same direction by up to four times, in agreement with experimental measurement of the response function. However, regardless of whether the direction change or not, a new speed and total run time are chosen from the same distributions, as above. Once a bacterium reaches an area 5 µm from the source of food at the corner it is removed from the system. This procedure continues for one hour.

The parameters used in these simulations were as follows:

1. The computational domain is 1mm × 1mm.
2. The cells/particles are initially distributed uniformly in the computational domain.
3. The time step is 0.1 s
4. New directions of runs are chosen from a uniform distribution in [0, 2π].
5. The background concentration of the attractant is given by:
  5 $$c(x,y)=\frac{A}{1+\exp [(r-0.4)/0.25]}+B$$
  where $r=\sqrt{{(1-x)}^{2}+{(1-y)}^{2}}$, and A and B are chosen so that $c(0,0)\,=\,10$ and $c(1,1)\,=\,100$.
6. The speed of the bacteria is assigned from a Lognormal distribution with $3\times {10}^{-2}$ mm/s, and a standard deviation of $8\times {10}^{-2}$ mm/s.
7. No matter how much the concentration increases between time steps, each bacterium has a probability of at least 0.25 of changing directions.
8. The number of time steps that a bacterium “runs” is distributed with a Poisson distribution with mean 10. Thus, the mean time that a bacterium runs is 1 s.
9. If a bacterium moves within 5×10^−3^ mm of the upper right corner, it is removed from the simulation.
10. If the bacteria secrete the chemical, it diffuses at a rate of *D* = 3 × 10^−4^ mm^2^/s.
11. The concentration of the secreted chemical is V = 5 × 10^−3^/m^2^.
12. The secretion is simulated with the two-dimensional heat kernel that is added to the background concentration. If a bacterium secretes from location $({x}^{\ast },{y}^{\ast })$ at time ${t}^{\ast }$, then for all later times $t > {t}^{\ast }$, the additional term

6 $$c(x,y)=\frac{V}{4\pi (t-{t}^{\ast })D}exp(\frac{-({(x-{x}^{\ast })}^{2}+{(y-{y}^{\ast })}^{2})}{4(t-{t}^{\ast })D})$$

is added to the external attractant gradient, where *V* is the amount that is secreted and *D* is the diffusion coefficient.

## Electronic supplementary material

Supplementary movie S1

Supplementary movie S2

Supplementary movie S3

Supplementary movie S4

Supplementary movie S5

Supplementary movie S6

Supplementary movie S7

Supplementary movie S8

Supplementary movie S9

Supplementary figures and movie captions
