# Supplementary material for: Cell-cell communication enhances bacterial chemotaxis toward external attractants
Source: Sci Rep. 2017 Oct 9;7:12855. doi: 10.1038/s41598-017-13183-9 (PMC5634484; doi:10.1038/s41598-017-13183-9)
Supplement: Supplementary file 10 — Supplementary figures and movie captions [file 41598_2017_13183_MOESM10_ESM.pdf]

Supplementary movies and figures

Cell-cell communication enhances bacterial chemotaxis toward external attractants

Zhicheng Long[1,2], Bryan Quaife[3], Hanna Salman[2,4] & Zoltán N. Oltvai[1,4]

Departments of [1]Pathology, [2]Physics and Astronomy, and [4]Computational & Systems Biology University of Pittsburgh, Pittsburgh, PA 15213, and [3]Department of Scientific Computing, Florida State University, Tallahassee, FL 32306

Supplementary movie S1: Wild-type RP437 cells swim into the chambers contained M9-G medium with (left chamber) or without (right chamber) 10μM Asp.

Supplementary movie S2: Wild-type cells migrate out of the chamber rapidly in Asp gradient.

Supplementary movie S3: Δ*tsr* mutant cells migrate out of the chamber slowly in Asp gradient.

Supplementary movie S4: Wild-type cells follow the gradient of the signaling molecule instead of aspartate.

Supplementary movie S5: Left panel shows a simulation of bacterial chemotaxis up an external attractant gradient starting with 1,000 cells in the chamber and without cell-cell communication. In the right panel we present the external attractant concentration as a function of position.

Supplementary movie S6: Same as movie S5 starting with 30 cells in the chamber.

Supplementary movie S7: Same as movie S6 with the cells secreting an attractant in response to sensing an increase in the external attractant concentration. The lower panels depict the external attractant (left) and the secreted attractant (right) concentrations as a function of position. The upper right panel depicts the combined attractant concentration (external + secreted) as a function of position.

Supplementary movie S8: Same as movie S7 starting with 1,000 cells in the chamber.

Supplementary movie S9: Same as movie S8 with the secretion being continuous and independent of the movement direction of the cell.

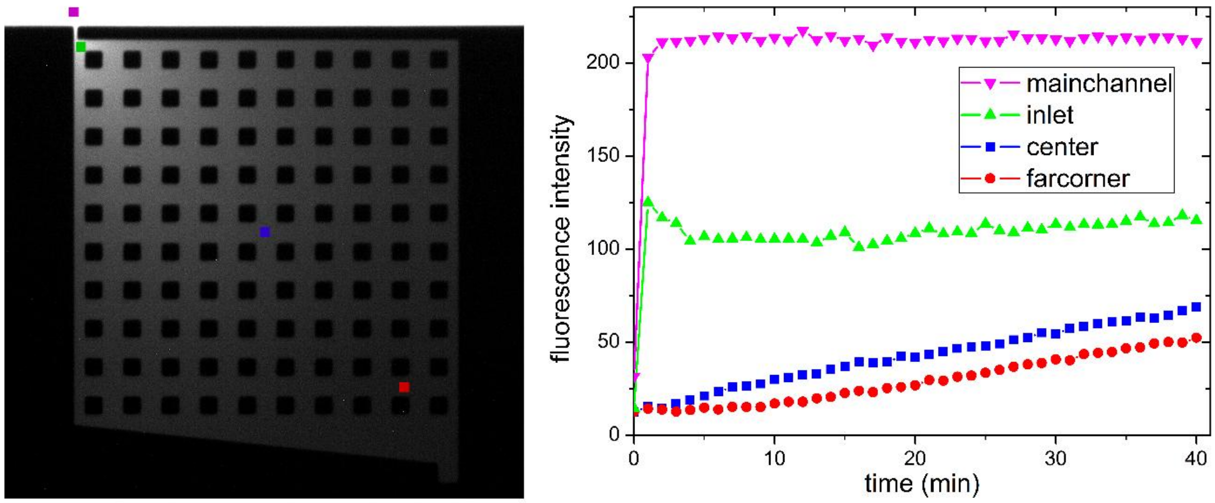

Figure S1 Fluorescence intensity of the gradient tracer dye

The fluorescence intensity of the gradient tracer dye, Alexa Fluor® 568 at the four positions marked on the left image changes with time. The measurements were from the same movie for Figure 1.

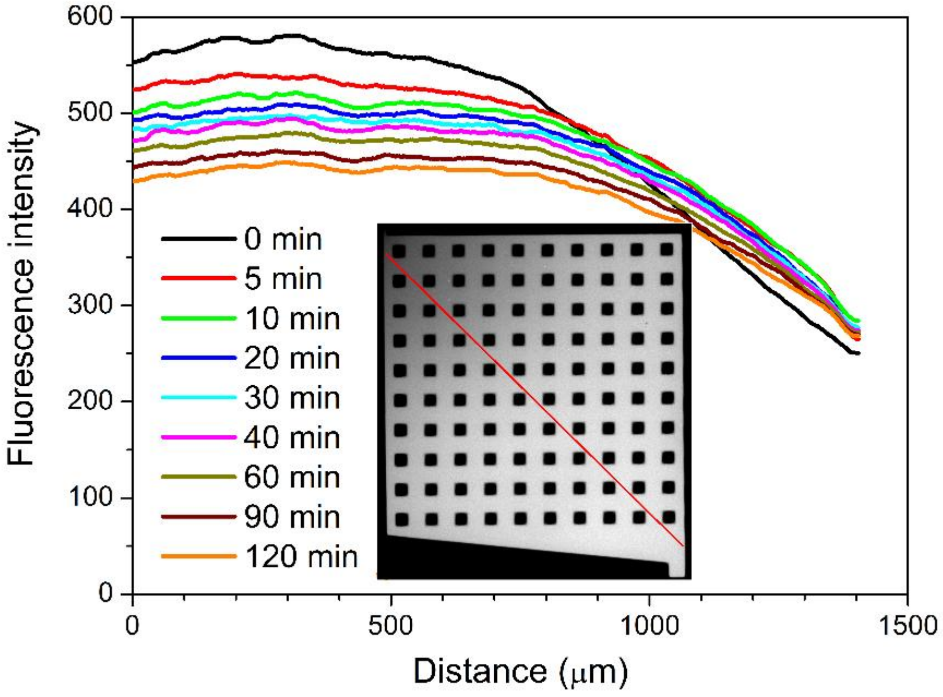

Figure S2 The gradient dynamics when there is no flow in the main channel

The gradient profiles along the red line (from the lower right nook corner to the upper left inlet) at various times. The microchamber was first filled with M9-G medium containing 10 µg/mL Alexa Fluor®568; the main channel was filled with non-fluorescent fresh medium containing *E. coli* cells and the flow inside the main channel was stopped.

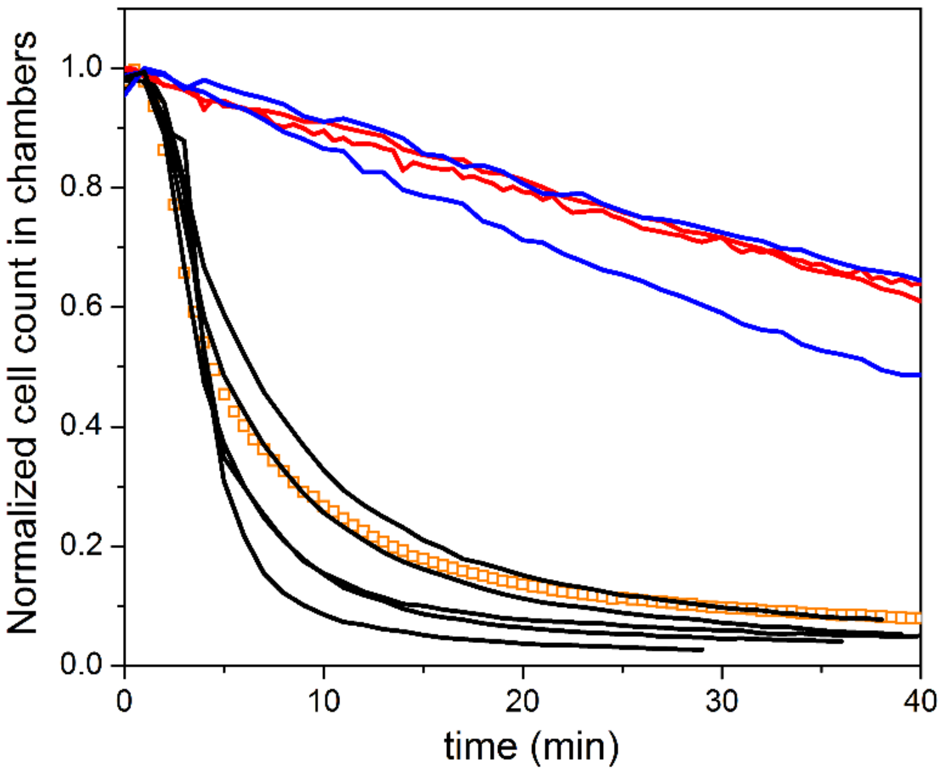

Figure S3 Wild-type and Δ*tar* cells show rapid migration in M9CG

The microchambers were filled with M9CG (the medium used for the blue curves also contained extra 10 mM serine) before loading the cells. Wt (black and blue curves), Δ*tar* (orange symbol) or Δ*tsr* (red curves) cells resuspended in fresh M9CG were then introduced into the main channel and swim into the microchambers. Fresh M9CG was pumped into the main channel after loading. Each curve is obtained from independent experiments performed on different days and shows the averaged result from 2 or 3 movies in the same experiment.

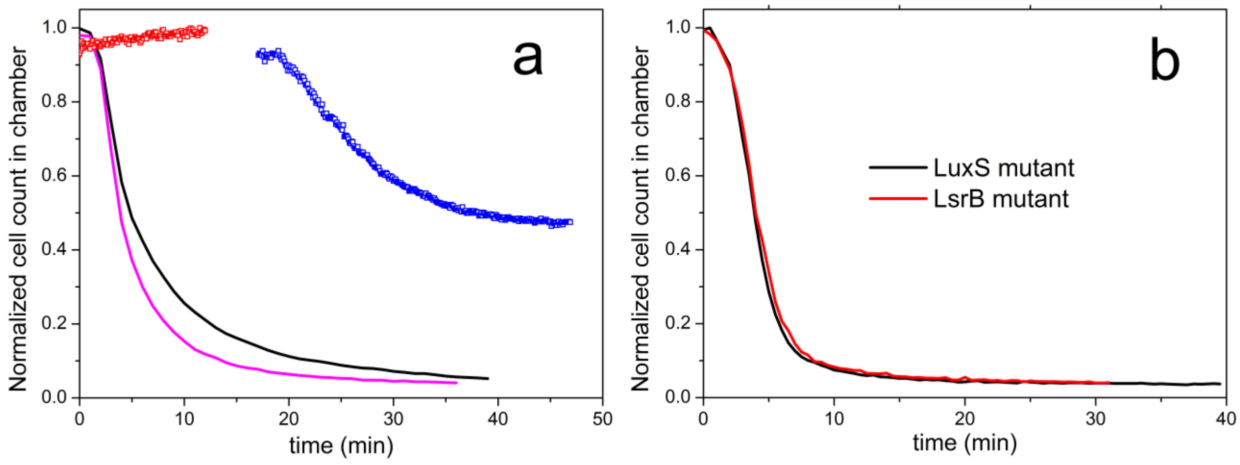

Figure S4 The signaling molecule is neither glycine nor auto inducer 2 (AI-2)

(a) The wild-type cells migrate out of the chamber rapidly in M9CG containing 10 mM glycine (solid curves). The chambers were filled with M9CG containing 10 mM glycine before loading the cells. Fresh M9CG was pumped into the main channel after loading. The red and blue scatter curves shows that the wild-type cells did not follow the gradient of glycine (red curve) but then migrated out rapidly in the gradient of Asp (blue curve). The chambers were filled with M9-G containing 10 µM Asp before loading the cells. Fresh M9G containing 100 µM glycine was firstly pumped into the main channel after loading. The media in the main channel was then changed to M9-G containing 200 µM Asp. (b) The *ΔluxS* (unable to produce AI-2) and *ΔlsrB* (unable to sense AI-2) mutant cells migrate out of the chamber rapidly in the gradient of Asp. The chambers were filled with M9-G containing 10 µM Asp before loading the cells. Fresh M9-G containing 200 µM Asp was pumped into the main channel after loading.

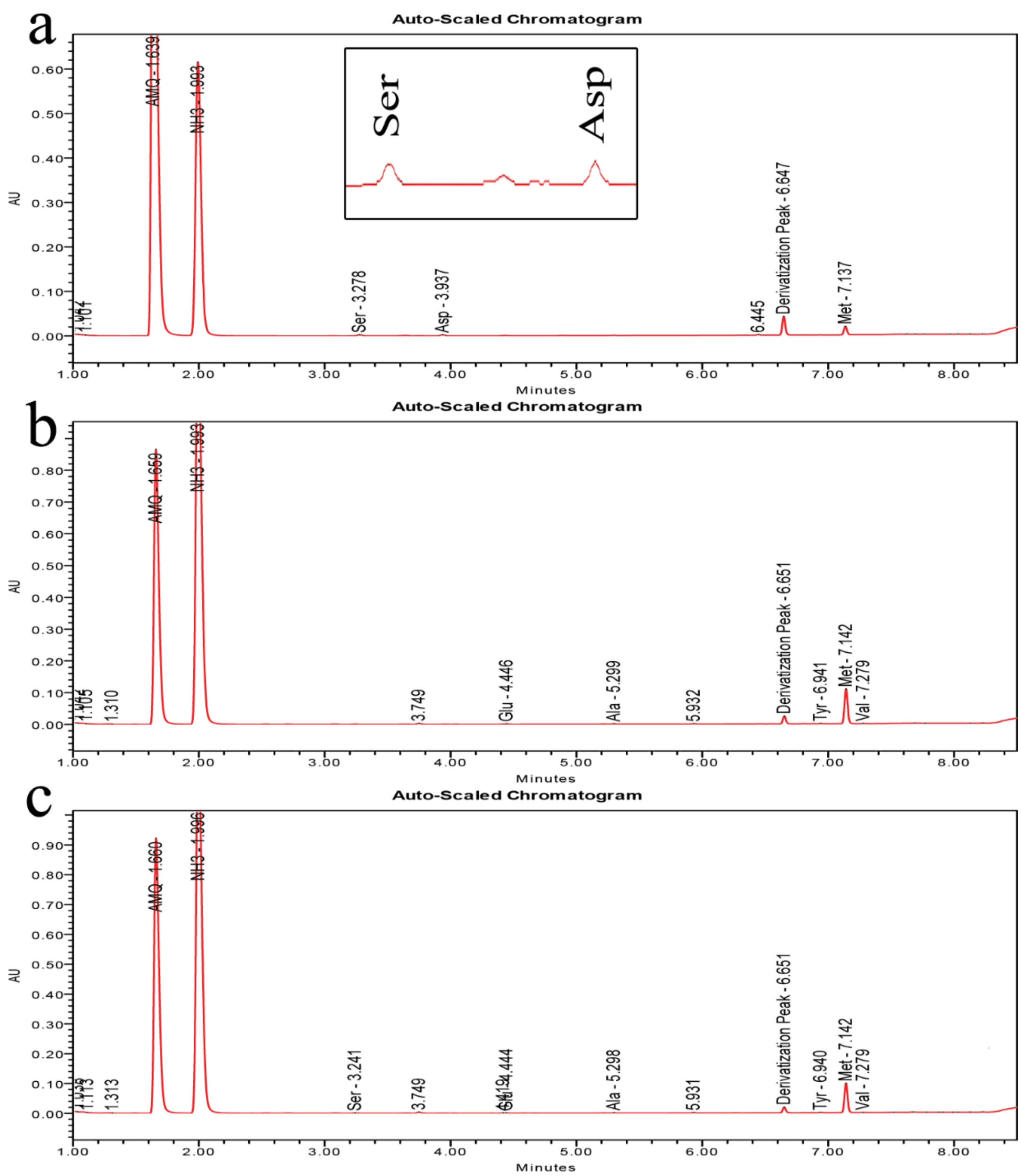

Figure S5 HPLC analysis of the free amino acids in extracellular medium

(a) M9-GA medium sample in which *wt* cells were exposed in Asp gradient for 20 minutes. (b, c) M9-GA medium sample in which *wt* cells were kept still for 120 min (b) and 240 minutes (c) in the absence of Asp gradient. Wild type *E. coli* cells were first grown in M9CG and then washed and resuspended in fresh M9-GA medium that does not contain any amino acids except 100 µM Asp and 20 µM methionine. The cells in the samples were removed immediately by centrifugation and the supernatants were filtered and kept frozen before HPLC analysis. Note that in the presence of an Asp gradient, serine appears in the medium after a short period of exposure (20 minutes compared with 240 without the gradient).
